# Endothelial cell control of thrombosis

**DOI:** 10.1186/s12872-015-0124-z

**Published:** 2015-10-19

**Authors:** Jonathan W. Yau, Hwee Teoh, Subodh Verma

**Affiliations:** Division of Cardiac Surgery, St. Michael’s Hospital, Suite 8-003, Bond Wing, 30 Bond St., Toronto, ON M5B 1W8 Canada; Divisions of Endocrinology & Metabolism, Keenan Research Centre for Biomedical Science at St. Michael’s Hospital, Toronto, ON Canada; Department of Surgery, University of Toronto, Toronto, ON Canada

**Keywords:** Endothelial cells, Thrombosis, Blood coagulation

## Abstract

Hemostasis encompasses a set of tightly regulated processes that govern blood clotting, platelet activation, and vascular repair. Upon vascular injury, the hemostatic system initiates a series of vascular events and activates extravascular receptors that act in concert to seal off the damage. Blood clotting is subsequently attenuated by a plethora of inhibitors that prevent excessive clot formation and eventual thrombosis. The endothelium which resides at the interface between the blood and surrounding tissues, serves an integral role in the hemostatic system. Depending on specific tissue needs and local stresses, endothelial cells are capable of evoking either antithrombotic or prothrombotic events. Healthy endothelial cells express antiplatelet and anticoagulant agents that prevent platelet aggregation and fibrin formation, respectively. In the face of endothelial dysfunction, endothelial cells trigger fibrin formation, as well as platelet adhesion and aggregation. Finally, endothelial cells release pro-fibrinolytic agents that initiate fibrinolysis to degrade the clot. Taken together, a functional endothelium is essential to maintain hemostasis and prevent thrombosis. Thus, a greater understanding into the role of the endothelium can provide new avenues for exploration and novel therapies for the management of thromboembolisms.

## Background

The endothelium has been described as a cellophane type barrier that separates the blood from the surrounding tissue. Although the endothelium is less than 0.2 μm thick, it is comprised of 1 to 6 × 10^13^ endothelial cells with a total surface area of 4000–7000 m^2^ and weighing approximately 1 kg in an average-sized human [1]. Instead of playing merely a passive role, the endothelium is better described as a dynamic organ that regulates its environment and responds to external stresses. Some of the functions of the endothelium include regulating vascular tone, cellular adhesion, smooth muscle cell proliferation, and vessel wall inflammation. In addition, the endothelium serves as a hemocompatible lining that helps to maintain blood flow and regulate the blood coagulation system. Upon vascular injury, cellular and protein materials congregate at the site of injury to create a stable blood clot, which prevents excessive blood loss. The significance of appropriate blood coagulation control is apparent during the development of thrombosis. Thrombosis is the formation of an occlusive clot within a blood vessel that reduces blood flow to distal tissue and organs and restricts the delivery of nutrients and oxygen, resulting in localized tissue and organ necrosis. Large occlusive clots (thrombi) can break off and embolize to form secondary thrombi in distal locations. The process of thrombosis followed by embolism is collectively termed, thromboembolism and can culminate in a variety of local or chronic disorders. For example, acute arterial thrombosis is triggered upon the rupture of an atherosclerotic plaque, and is the predominant cause of myocardial infarctions (heart attacks) and strokes [2]. Similarly, venous thromboembolism can be triggered by disturbed blood flow, hypercoagulable conditions, such as procoagulant changes in the blood, or endothelial activation, and is the major cause of deep vein thrombosis and pulmonary embolism [2, 3]. Since the endothelium is located at the nexus of hemostasis and thrombosis, a clear understanding of how endothelial-derived molecules contribute to hemostasis and thrombosis is vital for the identification of potential therapeutic targets and design of novel therapies for acute thrombosis.

## General functions of the endothelium

Endothelial cells have a myriad of functions that are specific to their location; they exhibit considerable phenotypic heterogeneity across different species and possess features that are distinct to each vascular bed [4, 5]. The main function of the endothelium is to regulate systemic blood flow and tissue perfusion through changes in vessel diameter and vascular tone, performed in conjunction with underlying smooth muscle cells and pericytes. In addition to regulating systemic blood flow, the endothelium acts as a barrier that selectively controls the movement of fluid, ions and other macromolecules between the circulating blood and surrounding tissues by coordinating the interendothelial adherens and tight junction complexes [6]. While serving as a selective barrier, the endothelium also regulates the recruitment and extravasation of pro-inflammatory leukocytes in response to tissue damage and infection through expression of cell adhesion molecules and cytokines [7, 8]. Endothelial cells have a critical role in the healing process after wounding or inflammation. They act as the vector of angiogenesis, the formation of new blood vessels, which is essential for proper formation of granulation tissue and tissue repair as well as for re-canalization of mural and obstructing fibrin clots. Finally, the luminal surface of the endothelium expresses a plethora of molecules that regulate the activation of platelets and the coagulation cascade, thereby maintaining blood flow and preventing thrombus formation after vessel injury [9, 10].

## Vessel- and tissue-specific functions of endothelial cells

Endothelial cells line the vessel walls of arteries, veins, and microvessels. The local shear stress, blood oxygenation, and smooth muscle cell content vary between these vessels and consequently the endothelial cells in the various vascular beds respond differentially to procoagulant signaling. Endothelial cells are dynamic and adapt their phenotype according to the nature of the local milieu [11, 12]. For example, vascular shear stress can influence the coagulant potential of endothelial cells. Arterial shear stress can induce the transcription factors Kruppel Like Factor (KLF) 2 and KLF 4 and attenuate coagulation in atherosclerosis-poor regions, but has no effect in atherosclerosis-prone areas with disturbed blood flow [13, 14]. Likewise, reduced venous shear stress can induce hypoxia and stimulate the release of P-selectin and von Willebrand factor from endothelial cells. The nature of the shear stress also has a significant influence on the type of thrombi that forms. Arterial clots form under high shear stress after atherosclerotic plaque rupture and are rich in platelets, giving the appearance of a white clot. In contrast, venous thrombi develop under low shear stress and are rich in fibrin and red blood cells, giving the appearance of a red clot. Venous thrombosis is particularly prevalent in the lower limbs, due to a variety of circumstances, including disturbed flow or local hypoxia. Of note, venous thrombi are exceptionally prone to embolism due to high availability of the fibrinolysis regulator, tissue-type plasminogen activator (t-PA) in the venous system. Microvascular endothelial cells within arteries and veins have distinctive features [15]. Surface receptors, such as thrombomodulin, while abundantly present in many types of endothelial cells, are poorly expressed or absent in brain microvascular and liver sinusoidal endothelial cells [16]. Microvascular endothelial cells also possess extensive phenotypic heterogeneity depending on their location [17]. Finally, the location of the endothelial cells also plays a role in the endothelial response. For example, pulmonary endothelial cells experience rapid changes in pressure from low-pressure, high-volume during gas exchange to high-pressure, low volume bronchial circulation during oxygen delivery to the bronchial tree. Moreover, pulmonary capillary endothelial cells are exposed to the highest level of oxygen compared with other vascular beds in the body. Taken together, although there are a wide variety of endothelial cells, they are similar in that they are all capable of evoking an array of responses that is based on cues provided by the local environment.

## The blood coagulation system

In the face of vascular injury, a chain reaction of pro-inflammatory and wound-healing responses is rapidly triggered. As part of the wound-healing response, a stable blood clot, consisting of aggregated platelets and a mesh of cross-linked fibrin protein, is formed to prevent excessive blood loss [18]. Traditionally, blood coagulation is described as occurring in two phases, primary and secondary hemostasis, wherein platelets first aggregate to form the initial platelet plug followed by activation of the coagulation system to form the fibrin clot. Depictions of blood coagulation have evolved significantly and now showcase the complex interplay between platelets, the coagulation system, and vessel wall to clot formation. This intricate network can be described as the cell-based model of coagulation. In short, blood coagulation is categorized into three phases: initiation, amplification, and propagation. Initiation occurs upon vascular injury with resultant activation of the endothelium, consisting of activated endothelial cells, and exposure to sub-endothelial cells that include amongst others smooth muscle cells and fibroblasts. Sub-endothelial collagen is exposed to the blood and mediates the initial adhesion of circulating platelets to the exposed collagen surface via von Willebrand factor. The platelet aggregate that forms is responsible for stopping blood loss. In parallel to these events, activated endothelial cells and smooth muscle cells express the potent pro-coagulant molecule, tissue factor (TF), which binds with circulating coagulation factor (f) VII. TF acts as a cofactor for fVII to promote the proteolysis and activation of fVII to become active fVII (fVIIa), wherein TF also binds with fVIIa to form the TF/fVIIa complex. The TF/fVIIa complex then goes on to proteolytically cleave fIX and fX into fIXa and fXa, respectively. FIXa serves to generate more fXa and fXa serves to generate thrombin. In the amplification phase, circulating platelets that have adhered to the site of injury become activated by thrombin and form a platelet aggregate. This provides a surface for the activation of other procoagulant factors. Concomitantly, thrombin proteolytically cleaves platelet-derived fV and circulating fVIII into fVa and fVIIIa, respectively. In addition, thrombin converts fXI into fXIa, which promotes further fIXa generation. In the final propagation phase, thrombin generation is amplified on the surfaces of activated platelets. FVa binds with fXa to form the prothrombinase complex, whereas fVIIIa binds with fIXa to form the intrinsic tenase complex. The prothrombinase and intrinsic tenase complexes serve to enhance the activities of fXa and fIXa, generating sufficient quantities of thrombin to produce large amounts of insoluble fibrin. In this phase, thrombin also cleaves fXIII into fXIIIa, which covalently cross-links fibrin chains to form a large fibrin mesh. Taken together, the platelet aggregate and cross-linked fibrin forms a stable clot, which seals off the site of injury and prevents excessive blood loss.

**Fig. 1:**
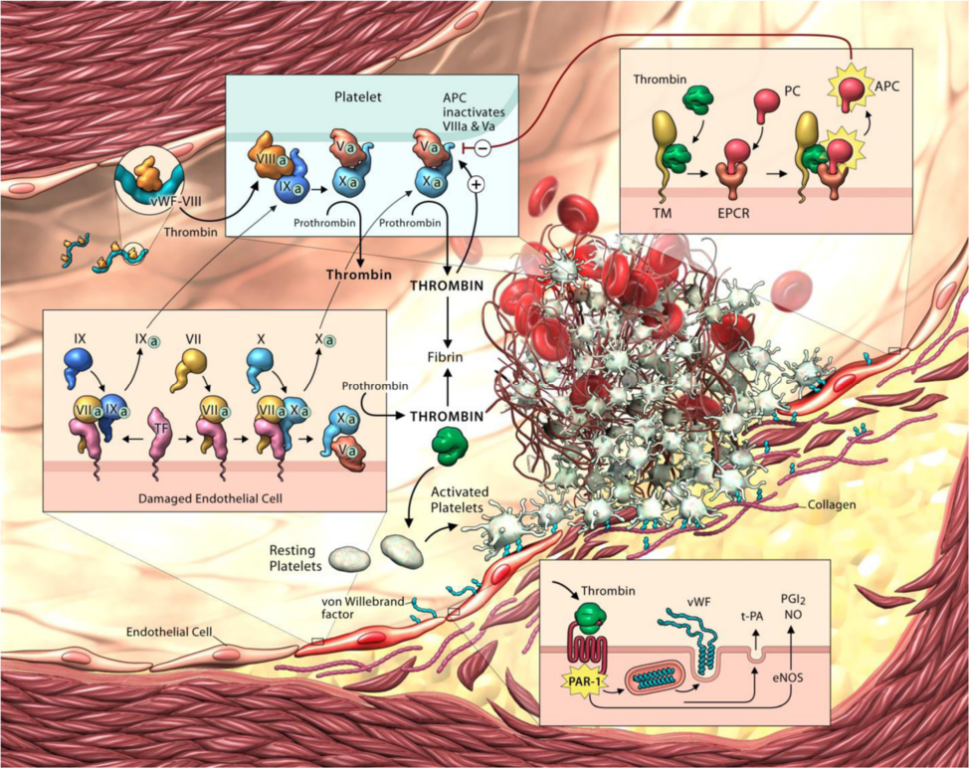
Illustration of endothelial control over blood coagulation

Since the blood coagulation system is a potent, highly effective process, tight regulation of the blood coagulation system is essential to prevent unnecessary clot formation [18]. Any perturbations of the regulatory pathways can accordingly culminate in thrombosis. Coagulation proteases and molecules can be inhibited by either direct inhibition of protease activity or through degradation of coagulation factors. First, circulating protease inhibitors, such as antithrombin, heparin cofactor II, tissue factor pathway inhibitor (TFPI), and C1 inhibitor, bind with the active site of proteases and prohibit the protease from cleaving its target. Table 1 indicates the respective targets for these inhibitors. Second, coagulation factors can be degraded through activation of the protein C/protein S pathway, activation of a disintegrin and metalloproteinase with a thrombospondin type 1 motif member 13 (ADAMTS13), or activation of tissue-type plasminogen activator (t-PA). The protein C/protein S pathway inactivates fVa and fVIIIa and is catalyzed by the presence of thrombomodulin and endothelial protein C receptor (EPCR). ADAMTS13 is a matrix metalloproteinase that cleaves the multimeric strands of von Willebrand factor, disrupting platelet adhesion. t-PA contributes to the final dissolution of the fibrin-mesh by triggering fibrinolysis through the conversion of plasminogen to plasmin. Collectively, these individual events come together to ensure that the blood coagulation system is highly regulated. As a result of its location, the endothelium plays an essential role in the development of a clot. Figure 1 illustrates the contribution of the endothelium to blood coagulation. The following sections provide an in-depth review of some of the key components of the blood coagulation system that originate from the endothelium.

**Table 1:** Targets of coagulation inhibitors

Inhibitor	Principal target(s)
Antithrombin	Thrombin, fXa, fIXa, fXIa, fXIIa
TFPI	fXa, TF/fVIIa
Heparin cofactor II	Thrombin
C1 inhibitor	fXIa, fXIIa, kallikrein
Protein C pathway	Thrombin

*Abbreviations*: *TFPI* tissue factor pathway inhibitor

### Endothelial contribution to the blood coagulation cascade

**Fig. 2:**
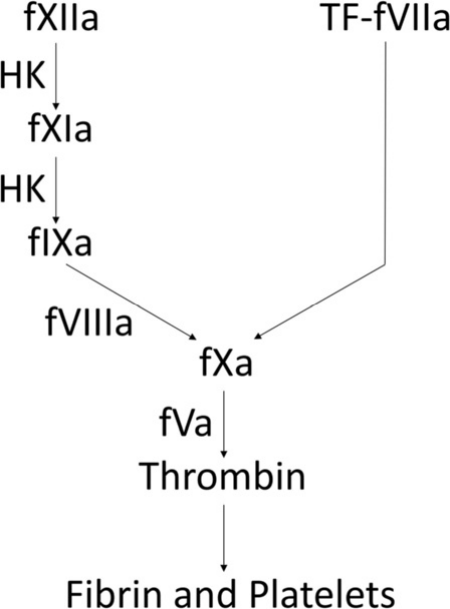
Succinct schematic overview of the blood coagulation cascade. *f* factor, *HK* high-molecular-weight kininogen, *TF* tissue factor

Traditionally, the blood coagulation system is described as a “cascade” or “waterfall” of Ca^2+^-dependent activation steps that begin with activation of the intrinsic pathway or extrinsic pathway and culminate in the production of the penultimate enzyme, thrombin (Fig. 2) [19, 20]. While the blood coagulation cascade model admittedly provides an over-simplified view of blood coagulation, it does highlight all of the essential protein components and is a useful tool for quickly understanding this otherwise rather intricate system. Intact endothelial cells express potent inhibitors, discussed in later sections, to prevent the synthesis and activity of thrombin. Once endothelial cells become activated, they play an essential role to the generation of thrombin through expression of pro-coagulant factors that contribute to both initiation and propagation of thrombin generation.

In the blood coagulation cascade, thrombin generation is initiated by activation of the extrinsic pathway, triggered by activation of fVII to form fVIIa, or by activation of the intrinsic pathway, triggered by activation of fXII to form fXIIa. In the extrinsic pathway, activated endothelial cells express TF on the cell surface. TF is constitutively expressed in extravascular tissues, such as fibroblasts and smooth muscle cells [21] and the phenomenon has been described as a hemostatic envelope that limits bleeding after vascular injury [22]. Although TF is not typically expressed in the intravascular space, activated endothelial cells and adhered leukocytes may express active TF in response to vascular injury or inflammatory stimuli. For example, activated endothelial cells express TF, which has an important role in the pathogenesis of thrombosis [23, 24]. TF is also abundantly expressed in atherosclerotic plaques and is found in both cellular (macrophages, vascular smooth muscle cells, and endothelial cells) and acellular (foam cell-derived debris within the necrotic core) regions [25, 26]. Furthermore, under experimental conditions, cultured endothelial cells express TF in the presence of pro-inflammatory molecules, such as lipopolysaccharide, tumor necrosis factor-α (TNF-α), interleukin-1β (IL-1β), thromboxane A2, vascular endothelial growth factor, and thrombin [27–40]. In contrast, only a few studies have reported endothelial TF expression in animal models. While some investigators have reported the presence of TF protein on the endothelial cells of lipopolysaccharide-treated mice and rabbits [41, 42], others have been unable to reproduce the results in similar models [43, 44]. These conflicting observations may in part have arisen from the different detection methods applied. Alternatively, TF may exist as an inactive (encrypted) form that becomes activated (decrypted) upon vascular injury [45] and this may account for the presence of TF protein with little to no procoagulant activity. How and why TF encryption occurs has been a matter of great debate [45], and is beyond the scope of this review. Several lines of evidence have suggested that the effects of endothelial cell-derived TF may not be confined to the coagulation network. The TF/fVIIa complex activates protease activated receptor (PAR)-2 [24], which induces a pro-inflammatory response. TF deficiency has been associated with decreased IL-6 expression via fXa-dependent activation of PAR-2 in a murine model of sickle cell disease [46]. Furthermore, microvascular endothelial cells have been observed to induce angiogenesis and collateral vessel formation through the release of TF-rich microparticles [47]. The collective data suggest that TF has a multitude of hematologic and vascular effects although further work is required to elucidate the origin and role of endothelial cell-derived TF.

While activated endothelial cells are typically associated with the extrinsic pathway, they may serve additional roles with the intrinsic pathway. For example, cultured endothelial cells induce thrombin generation in platelet-poor plasma. The presence of a TF inhibitor has a limited effect on thrombin generation whereas low levels of thrombin are formed in fXII-deficient plasma. Similarly, thrombin generation is enhanced in patients undergoing coronary artery bypass grafting, suggesting that the intrinsic pathway may be important under specific conditions. The underlying mechanisms of how endothelial cells affect the intrinsic pathway is currently unclear although it is plausible that endothelial cells may in part act as a barrier for intrinsic factors from inhibition since fXIIa is protected from inhibition by C1 inhibitor in the presence of endothelial cells [48]. Thus, endothelial cells may be critical components of both the intrinsic and extrinsic pathways.

Once the blood coagulation cascade is triggered, thrombin is generated and amplifies its own production through activation of cofactors fV and fVIII. While fV, as well as most other pro-coagulant proteins, is derived from hepatocytes, the origins of fVIII have been elusive. An early study by Webster et al. demonstrated that canines with fVIII deficiency (Hemophilia B), that received liver transplants from normal canines, exhibited reduced bleeding tendencies compared with non-transplanted canines [49]. Despite reduced bleeding tendencies, fVIII deficient canines possessed 50 % of the fVIII plasma levels compared with normal canines. Similarly, normal dogs who received liver transplants from hemophilic dogs also maintained a fVIII level of 50 %. Follow-up investigations in in vitro settings have provided evidence suggesting that liver sinusoidal endothelial cells, and not hepatocytes, produce fVIII in culture [50]. Extra-hepatic endothelial cells may also synthesize fVIII. Norman et al. and Veltkamp et al. demonstrated that fVIII deficient animals who received spleen or lung transplantations had partially restored levels of fVIII in plasma [51, 52], suggesting that extra-hepatic sources of fVIII existed. Fahs et al. demonstrated that mice with hepatocyte-specific deletion of fVIII were indistinguishable from littermate controls. In contrast, mice with endothelial-specific deletion of fVIII displayed a severe hemophilic phenotype with no detectable levels of plasma fVIII activity [53]. Everett et al. also demonstrated that endothelial-specific deletion of *Lman1* in mice had lower levels of plasma fVIII compared with their control group [54]. Conversely, hepatocyte-specific deletion of *Lman1* in mice did not reveal any effect on plasma fVIII levels, supporting the observation that fVIII is synthesized in multiple types of endothelial cells. Together, these results provide solid evidence that endothelial cells are the primary source of fVIII.

### Endothelial contribution to platelet adhesion and aggregation

Platelets play a fundamental role to prevent blood loss by forming the platelet hemostatic plug, and to serve as a platform for coagulation factors. Platelet-endothelium interactions play an integral role in the activation and regulation of platelets. While an intact endothelium inhibits the adhesion of platelets, through the release of nitric oxide and prostaglandin I_2_, activated endothelial cells express a variety of molecules and receptors that increase platelet adhesion to the site of injury. In endothelial cells, Weibel-Palade bodies store VWF, P-selectin, angiopoietin-2, t-PA, and endothelin-1, which are active participants of platelet adhesion, leukocyte recruitment, inflammation modulation, fibrinolysis, and vasoconstrictor, respectively. Following vascular insult or in the presence of vasoactive agents such as histamine, bradykinin, and thrombin, endothelial Weibel-Palade bodies fuse with the plasma membrane and release these products into the abluminal space wherein they perform their respective functions.

VWF, a predominant product of the endothelium, is a multimeric adhesion glycoprotein. It is synthesized within the endoplasmic reticulum and processed by the Golgi complex of endothelial cells, megakaryocytes, and the α-granules of platelets [55]. In hemostasis, VWF plays a dual role. First, VWF is essential for platelet adhesion to collagen at sites of vascular injury. Following vascular insult, VWF forms long strings of up to several millimeters in length on the surface of endothelial cells resulting in ultra-large VWF multimers that readily form high-strength bonds with platelets via the platelet receptor glycoprotein Ib-V-IX, and stretch along the surface of the endothelium or of the damaged vessel. The second function of VWF is to stabilize circulating plasma fVIII [56]. VWF stabilizes fVIII through a non-covalent interaction, which results in a tightly bound complex [57]. The importance of VWF in fVIII stabilization is demonstrated by the rapid clearance of fVIII from the circulation, resulting in a moderate hemophilia-like phenotype [58, 59]. Because of its dual role, VWF is an important contribution to hemostasis by endothelial cells.

VWF secretion has been described as constitutive, constitutive-like, and regulated, whereby an agonist acts as the trigger [60–62]. Constitutive secretion occurs when pro-VWF is released from resting endothelial cells via a canonical constitutive secretory pathway. Constitutive-like, or basal, secretion of VWF occurs when VWF is released from storage granules without stimulation. Despite extensive research, the exact mechanism by which VWF is secreted remains poorly defined. Several studies have suggested that VWF secretion may be dependent on different molecular pathways. In mice with endothelial cell specific knockout of G protein subunits (Gα12 and Gα11), thrombin-induced secretion of VWF was reduced whereas basal secretion of VWF was decreased in Gα12 knockout mice [63], suggesting that VWF secretion is G protein dependent. Another study implicated autophagy, an intracellular process, as a regulator of VWF secretion [64]. In mice with endothelial cell specific knockout of *ATG7*, a critical component of autophagy, VWF secretion is decreased and maturation of VWF is hindered. In addition, these mice have prolonged bleeding times compared with control. Interestingly, secretion of VWF is also influenced by circulating sodium levels [65]. In mice subjected to water restriction, protein levels of VWF in the endothelium rose along with an increased number of microthrombi inside capillaries [65].

### Endothelial regulation of the blood coagulation system

Under physiological conditions, the endothelium prevents thrombosis by providing a surface that discourages the attachment of cells and clotting proteins [66]. The endothelium regulates clot formation in part via its activation of the intravascular PARs. PARs exist as four isoforms, PAR-1, PAR-2, PAR-3, and PAR-4, and are expressed in arterial and venous endothelial cells [67–69]. Acute release of endothelial products in coagulation is largely mediated via PAR-1 [70]. Endothelial PARs serve as sensors for proteases and initiate a cascade of cell signals upon activation by thrombin, APC, fXa, the TF/fVIIa/fXa complex, high concentrations of plasmin, and matrix metalloproteases [69–71]. First, thrombin-mediated activation of PAR-1 is responsible for the production of nitric oxide and prostacyclin, which limits platelet activation. Second, thrombin-mediated activation of PAR-1 induces the activation of Weibel-Palade bodies, releasing VWF and t-PA. Lastly, thrombin-mediated activation of PAR-1 mediates the surface exposure of TF. Taken together, PAR-1 plays an important role in the pro-coagulant response upon stimulation.

An intact and healthy endothelium expresses various anticoagulants, such as TFPI, thrombomodulin, EPCR, and heparin-like proteoglycans [72]. Endothelial cells also secrete ectonucleotidase CD39/NTPDase1, which metabolizes the platelet agonist ADP, and platelet inhibitors, such as nitric oxide and prostacyclin [66]. As such, the endothelium actively regulates the powerful coagulation response through equally potent inhibitory processes.

One of the most important endothelium-derived inhibitors of the blood coagulation cascade is TFPI. A detailed overview of the structure and function of TFPI is extensively reviewed elsewhere [73]. Briefly, TFPI is a Kunitz-type protease inhibitor that inhibits the coagulation cascade by direct inhibition of free fXa and the TF/fVIIa/fXa complex. TFPI is present in endothelial cells but can also be found in megakaryocytes and platelets [73], and can be released upon treatment with heparin [74–76]. While TFPI exists as three isoforms (α, β and δ), TFPIα and TFPIβ are the predominant isoforms. TFPI also possesses a cofactor, Protein S. Protein S aids in the TFPI-induced inhibition of fXa and stabilizes the TFPI/fXa inhibitory complex thereby delaying thrombin generation by prothrombinase [77]. However, the catalytic activity of protein S is limited to platelet- and endothelial cell-derived TFPIα whereas protein S has no effect on cell surface-associated TFPI [77]. The importance of TFPI in hemostasis has been made very apparent in in vitro and animal models. Early studies demonstrated that inhibition of TFPI decreases the time to clot in normal plasma, suggesting that TFPI is an important regulator of coagulation. Mice with systemic homozygous deletion of the first Kunitz domain of TFPI (TFPI^tm1Gjb^; tfpi^−/−^) suffer from intrauterine lethality due to coagulopathy [78]. Those with hematopoietic-specific deletion of TFPI experience increased clot volume following vascular injury, whereas mice with endothelial-specific deletion of TFPI have decreased time to vascular occlusion following ferric chloride-induced injury [79]. Complete TFPI deficiency is a condition that has not been reported in humans, likely due to embryonic lethality. Patients with TFPI levels at or less than the 10th percentile of the normal reference range for TFPI are at slightly increased risk for venous thrombosis and coronary heart disease [80, 81]. This corroborates the notion that endothelium-derived TFPI is important for prevention of thrombosis.

Once the cascade is initiated, thrombin generation is amplified in the presence of activated cofactors. Since fVa and fVIIIa are essential cofactors for the amplification of thrombin production, their inactivation provides an avenue for regulating the blood coagulation cascade. This can be achieved through thrombin-mediated activation of the protein C pathway, which is catalyzed by endothelial cells via thrombomodulin and EPCR. Thrombomodulin is a 60-kDa transmembrane protein that is predominately synthesized by endothelial cells and expressed in all tissues, except for the microvasculature of the brain. Its transcription is regulated by shear stress and involves the transcription factor KLF-2 [82]. Thrombomodulin has been implicated in coagulation, inflammation, cancer development, and embryogenesis. Evidence to date suggests that thrombomodulin possesses three independent anticoagulant activities: catalyzing thrombin-induced activation of protein C to activated protein C, binding with thrombin to prevent conversion of fibrinogen to fibrin and activation of platelets, fV, fVIII, fXI, and fXIII, and catalyzing the inhibition of thrombin by antithrombin [83]. As the concentration of thrombin rises during coagulation, thrombomodulin forms a complex with thrombin to create the thrombin-thrombomodulin complex that proteolytically activates protein C and generates activated protein C, which confers anticoagulant activities [84]. Activated protein C degrades fVa and fVIIIa, and thus, attenuates thrombin generation. The thrombin-thrombomodulin complex enhances the conversion of protein C to activated protein C by 1000-fold compared with thrombin alone [85]. Although thrombin-thrombomodulin-mediated protein C activation is relatively efficient in the microvasculature, protein C cleavage in larger vessels requires the presence of EPCR, which is primarily localized on the endothelial layer of larger vessels [16]. EPCR is a type 1 transmembrane glycoprotein that possesses a similar homology to the major histocompatibility complex-class 1/CD1 family of molecules [86]. EPCR recruits protein C at the endothelial cell surface thereby facilitating the interaction between protein C and the thrombin-thrombomodulin complex. In the presence of EPCR, the conversion of protein C to activated protein C is increased by 20-fold compared with the thrombin-thrombomodulin complex alone [87]. EPCR has also been shown to bind with activated protein C [88]. The importance of EPCR is highlighted in studies involving mice. Mice with total deficiency in EPCR die in utero [89], suggesting that EPCR is not only important for suppression of thrombosis but is essential for normal embryonic development. Taken together, thrombin-mediated activation of protein C requires the presence of thrombomodulin and EPCR to efficiently generate activated protein C.

### Endothelial contribution to clot resolution

Once the wound is repaired, endothelial cells release pro-fibrinolytic molecules to degrade the clot and metalloproteases to cleave platelet aggregates. Dissolution of the clot is induced by fibrinolytic agents, such as t-PA and urokinase-type PA (u-PA). Although t-PA and u-PA perform the same function, t-PA is predominantly found in endothelial cells while u-PA is expressed in endothelial cells, macrophages, renal epithelial cells and some tumor cells. For simplicity, we primarily focus on t-PA since endothelial cells are the primary source of t-PA. t-PA is a 70-kDa protein that catalyzes the degradation of fibrin within the clot [90]. t-PA performs this function by activating the fibrinolytic system through conversion of plasminogen to plasmin in blood and body cavities [91]. In cultured endothelial cells, there is evidence that t-PA is stored in two types of storage granules: Weibel-Palade bodies [92] and small storage granules [93]. After wound repair is complete, the high local increase in t-PA at the site of thrombin generation allows for efficient removal of fibrin deposits at the luminal side of an intact vascular endothelium and is important for reducing tissue damage. The importance of t-PA has been demonstrated in t-PA deficient mice wherein clot lysis was impaired, especially when combined with u-PA deficiency [94, 95]. In experimental primate models of acute disseminated intravascular coagulation, plasma concentrations of t-PA was acutely increased by more than two orders of magnitude, due to thrombin-mediated release from storage granules in endothelial cells [96]. Thus, t-PA provides an essential method for removal of blood clots.

In a similar fashion, dissolution of platelet aggregates is mediated by ADAMTS13 [97]. Endothelial cells produce and release functionally active ADAMTS13, a zinc/calcium VWF-specific metalloprotease, and are an important source of plasma ADAMTS13 [98]. The importance of ADAMTS13 is highlighted by the observation that patients with thrombotic thrombocytopenia purpura, the levels of ADAMTS13 is severely reduced or absent [99]. This lack of ADAMTS13 may be due to inadequate production/release of ADAMTS13 or the activity ADAMTS13 is inhibited by auto-antibodies.

### Endothelial contribution to tissue repair and angiogenesis

While the blood coagulation system forms the stable clot that stems the loss of blood, it also contributes toward subsequent healing processes such as tissue repair and angiogenesis. Since endothelial cells are important component of blood vessels, they can be triggered to induce angiogenesis upon stimulation. For example, in addition to its anti-coagulant abilities, activated protein C has been shown to stimulate angiogenesis in brain endothelium [100]. Second, cross-linked fibrin serves as a scaffold for endothelial cells to synthesize new blood vessels [101, 102]. Third, platelets contain a rich source of vasoactive agents and chemokines, such as serotonin, thromboxane A2, platelet activating factor and RANTES, and pro-angiogenic growth factors, such as VEGF, all of which are contained in platelet α-granules [103, 104]. These compounds stimulate endothelial cell proliferation and promote the growth of new blood vessels. Lastly, the expression of TF has been shown to induce tumor angiogenesis in colorectal cancer and breast cancer models through TF-fVIIa-dependent PAR-2 activation [105–107], which induces the expression of VEGF, IL-8, MMP-7, and CXCL-1. In the past 10 years, studies have highlighted the role of a TF isoform, alternatively spliced TF [108–110], which displays no pro-coagulant activity but may play a prominant role in promoting angiogenesis. Taken together, the role of the coagulation system plays a major role in the development of angiogenesis.

### Endothelial contribution to aberrant clot formation

The endothelium is an essential component of the blood coagulation system and necessary to maintain hemostasis. However, endothelial dysfunction can occur in response to a variety of conditions, including elevated cholesterol, diabetes or smoking, which can induce vascular inflammation. Endothelial dysfunction is responsible for inflammation and blood coagulation, and is associated with cardiovascular disease, such as hypertension, coronary artery disease, chronic heart failure, peripheral artery disease, diabetes, chronic kidney failure, and viral infections. During endothelial dysfunction, endothelial cells become activated and contribute to the pathogenesis of thrombosis. For example, hypoxic conditions often lead to endothelial dysfunction. As such, hypoxia has been shown to promote the release of VWF from Weibel-Palade bodies in endothelial cells. Inflammation has also been associated with endothelial dysfunction and can be accompanied by thrombosis. For example, in vitro and in vivo studies have demonstrated that pro-inflammatory cytokines, such as TNF-α and IL-1, up-regulate the production of TF and VWF, while attenuating the expression of thrombomodulin and nitric oxide and prostaglandin I_2_. In rabbits, immunodepletion of TFPI leads to increased susceptibility to disseminated intravascular coagulation (DIC) [111], and generalized Schwartzman reaction, characterized by fibrin deposition and hemorrhagic necrosis in kidneys following infusion of TF [112]. The Protein C pathway has also been implicated in the development of disseminated intravascular coagulation with associated organ dysfunction. Patients with systemic inflammation show an impaired protein C system due to impaired protein C synthesis and impaired protein C activation. While protein C is synthesized by hepatocytes, endothelial cells can regulate protein C activation through the expression of thrombomodulin. As such, thrombomodulin levels are significantly down-regulated by the presence of pro-inflammatory cytokines, such as TNF-α and IL-1, resulting in diminished protein C activation. These events result in a shift from anti-thrombotic to pro-thrombotic conditions.

## Conclusions

The endothelium is a dynamic lining that regulates the complex interplay of the coagulation system with the surrounding cells and tissues. When stressed beyond a certain threshold, the quiescent endothelium initiates numerous mechanisms to induce clotting (e.g. by expression of TF and fVIII) and anticoagulation (e.g. by expression of thrombomodulin, EPCR, and PAR-type receptors) (Table 2). Concomitantly, activated endothelial cells recruits platelets to the site of injury (e.g. by expression of VWF), where it serves as a support surface for formation of pro-coagulant complexes and platelet aggregation. The biochemical processes described above highlight the critical contributions of multiple endothelial molecules in hemostasis and thrombosis, thereby underscoring the central role of the endothelium in these processes.

**Table 2:** Endothelial products in coagulation

Protein/Receptor	Function
PAR	Induces release of nitric oxide and prostacyclin I_2_, activation of Weibel-Palade bodies, and expression of TF
TF	Triggers the production of thrombin through activation of fX when in complex with fVIIa
fVIII/fVIIIa	Amplifies the production of thrombin when in complex with fIXa
VWF	Tethers platelets to collagen via glycoprotein VI on platelets
	Stabilizes fVIII in plasma
TFPI	Inhibits the activity of the TF/fVIIa complex and fXa
Thrombomodulin	Eliminates the activity of thrombin when in complex
	Induces the activation of the protein C pathway
EPCR	Catalyzes conversion of Protein C to activated Protein C
t-PA	Triggers fibrinolysis by converting plasminogen to plasmin

*Abbreviations*: *EPCR* endothelial protein C receptor, *f* factor, *PAR* protease-activated receptor, *TF* tissue factor, *TFPI* tissue factor pathway inhibitor, *t-PA* tissue-type plasminogen activator, *VWF* von Willebrand factor

## Abbreviations

APC: Activated protein C
EPCR: Endothelial protein C receptor
f: Factor
KLF: Kruppel like factor
PAR: Protease-activated receptor
TF: Tissue factor
TFPI: Tissue factor pathway inhibitor
t-PA: Tissue-type plasminogen activator
ULVWF: Ultra-large VWF multimers
VWF: Von Willebrand factor
